# Chronic Alcohol Abuse-Induced Hypokalemia Might Lead to Delayed Diagnosis or Misdiagnosis of Thyrotoxic Periodic Paralysis

**DOI:** 10.7759/cureus.15880

**Published:** 2021-06-23

**Authors:** Yan-Yu Lin, Yu-Shan Hsieh

**Affiliations:** 1 Division of Endocrinology and Metabolism, Department of Internal Medicine, Taipei Medical University Hospital, Taipei, TWN; 2 School of Nursing, National Taipei University of Nursing and Health Sciences, Taipei, TWN

**Keywords:** thyrotoxic periodic paralysis, alcohol abuse, hypokalemia, graves' disease, endocrine, case report

## Abstract

Thyrotoxic periodic paralysis is an uncommon and potentially life-threatening complication of thyrotoxicosis and hyperthyroidism characterized by acute and reversible episodes of muscle weakness and hypokalemia. Here is a 41-year-old Taiwanese male patient without any family history of hyperthyroidism presented to the emergency room of our institution with initial symptom of acute lower limb weakness. Laboratory analysis revealed uncommonly severe hypokalemia (<1.5 mEq/L). A thyroid function test revealed hyperthyroidism, and thyroid ultrasonography revealed findings compatible with Graves’ disease. However, symptoms such as nausea, vomiting, diarrhea, and heavy breathing were absent. He was administered with 15 mg of methimazole and 30 mg of propranolol per day for complications of hyperthyroidism. Then we exhaustively evaluated the patient’s history and lifestyle habits, and found that the patient had chronic alcohol abuse (an 1-L bottle 45%-48% liquor per week) for more than 10 years. In this case, chronic alcohol abuse may have increased the patient’s tolerance to the profound hypokalemia such that it did not immediately show critical symptoms. Therefore, according to this case report, we suggest that chronic alcohol consumption or abuse may lead patients, especially those with hyperthyroidism, to ignore or delay treatment.

## Introduction

Thyrotoxic periodic paralysis (TPP) is an uncommon and a potentially life-threatening complication of thyrotoxicosis, characterized by acute and reversible episodes of muscle weakness and hypokalemia. Although the incidence is only approximately 2% in patients with hyperthyroidism, and the symptoms of paralysis arise within a few hours [1], some cases of ventricular arrhythmia still have been reported [2]. Hence, early diagnosis and prompt treatment are important for avoiding complications and mortality. However, among causes of hypokalemia in patients with hyperthyroidism, alcohol consumption is one potential but usually ignored cause. Hypokalemia is commonly found in 50% patients with chronic alcoholism [3]. It is associated with an increased risk of arrhythmia in patients and as much as a 10-fold increase in all-cause mortality [4]. A previous study found that hypokalemia was more common in patients who abused alcohol than in a control population [5].

Therefore, chronic hypokalemia [6] and even other electrolyte disorders [7] can be observed in patients with alcoholism. Here, we present an interesting case of TPP in a Taiwanese male patient who also had a history of chronic alcohol abuse for almost 10 years. The patient presented with a sudden onset of weakness in the bilateral lower extremities and uncommonly severe profound hypokalemia.

## Case presentation

A 41-year-old Taiwanese male patient without any family history of hyperthyroidism presented to the emergency room of our institution with an initial symptom of acute limb weakness (level of muscle power is 2-3). Laboratory analysis revealed uncommonly severe hypokalemia (<1.5 mEq/L); the serum potassium level was not even detectable.

However, symptoms, such as nausea, vomiting, diarrhea, heavy breathing, and EKG changes, were absent.

**Figure 1:**
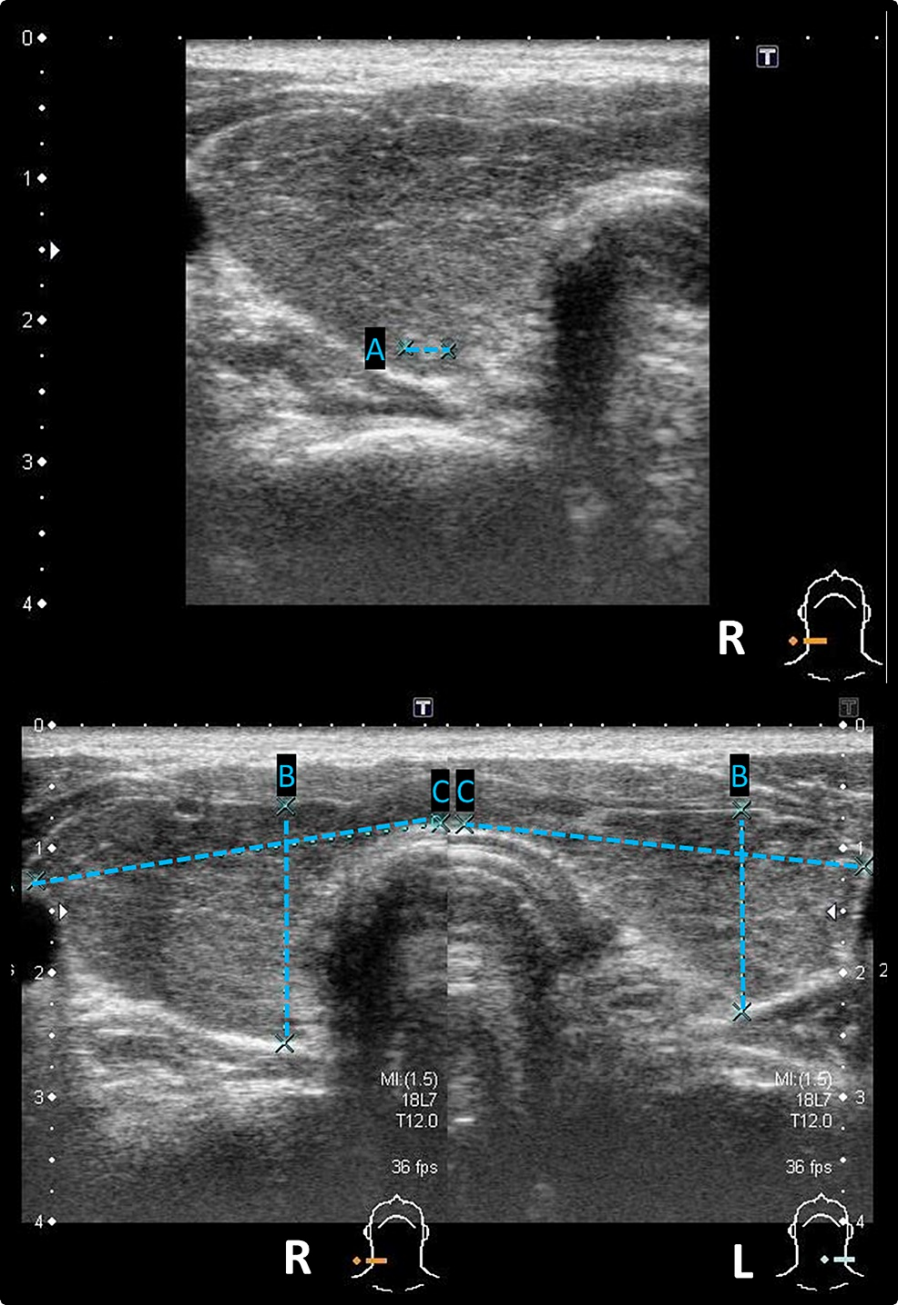
Thyroid ultrasonography A: Compatible with Graves' disease. Right 1 nodule: 0.31 cm; B, C: Size of thyroid, right side is 3.28 x 1.91 cm and left side is 3.22 x 1.62 cm.

There was no history of recent strenuous exercise, diuretic use, or other chronic disease, but the patient had chronic alcohol abuse (an 1-L bottle 45%-48% liquor per week) for more than 10 years. Other abnormal findings were observed: a thyroid function test (TFT) revealed hyperthyroidism with serum free thyroxine (fT4) level >6.60 ng/dL and a thyroid-stimulating hormone (TSH) level 0.06 mU/L. The serum TSH receptor antibody binding percentage was high at 85.92%, and thyroid ultrasonography findings were compatible with Graves' disease (Figure 1). These findings were consistent with Graves’ disease as the etiology of his hyperthyroidism.

The results of other biochemical studies are shown in Table 1. These showed profound electrolyte abnormalities such as hypokalemia. Due to the findings of severe hyperthyroidism and hypokalemia, he was admitted to the ICU for further care and he then received potassium replacement (potassium 10 mEq) acutely. He was administered 15 mg of methimazole and 30 mg of propranolol per day for complications due to hyperthyroidism. Following the treatment, the potassium level improved to 3.7 mEq/L. One month after treatment, a follow-up TFT revealed a near-normal fT4 (1.38 ng/dL) without rebound hyperkalemia. No paralysis occurred, and the hyperthyroidism was well controlled with continued anti-thyroid drugs and β-blockers prescribed in the outpatient department.

**Table 1:** Initial laboratory results of the patient WBC: white blood cell; RBC: red blood cell; HGB: hemoglobin; HCT: hematocrit; PLT: platelet; TSH: thyroid-stimulating hormone; fT4: free thyroxine; T3: triiodothyronine; TRAb: TSH receptor autoantibodies; OPD, outpatient department.

Laboratories (reference range)			Initial value	Interval value (after potassium supplementation)	Interval value (after propranolol)	Interval value (after anti-thyroid agent)	Interval value	Value prior to discharge	OPD follow-up value
Complete blood count									
	WBC (×10^3 ^µL)		4.93		12.51		6.88	6.85	
	RBC (×10^6 ^µL)		5.26		5.03		4.59	4.96	
	HGB (g/dL)		15.1		14.6		13.6	14.4	15.7
	HCT (%)		43.7		42.2		38.1	41.6	
	PLT (×10^3 ^µL)		370		328		322	421	
Blood gas									
	Venous pH (7.31-7.41)		7.533						
	Venous PCO_2_ (41-51 mmHg)		23.1						
	Venous HCO_3_ (19-26 mmol/L)		19						
Routine chemistry									
	Sodium (136-145 mEq/L)		144	141	139	140	141	141	
	Potassium (3.5-5.3 mEq/L)		<1.5 (undetectable)	4.4	5.9	5.3	4.5	3.7	
	Chloride (98-107 mEq/L)				109				
	Blood urea nitrogen (7-18 mg/dL)		15		16		14	14	
	Creatinine (0.7-1.20 mg/dL)		0.7		0.6		0.6	0.5	1
	Glucose (70-99 mg/dL)				137				
	Calcium (8.6-10.2 mg/dL)		9.8		9.2		9.5		
	Magnesium (1.6-2.6 mg/dL)		1.7		2		1.8		
	Phosphorous (2.7-4.5 mg/dL)				4.9				
	Troponin (0.00-0.014 ng/dL)		0.034	0.457	0.322	0.283	0.278	0.292	
	Creatinine kinase (20-200 U/L)		162	478	393	331	133	53	
Urine test									
	Spot urine potassium (none, mEq/L)			8					
	Spot urine creatine (25~259 mg/dL)			79.3					
Evaluation of thyroid function									
	TSH (0.4-5.0 µU/mL)		0.06			<0.05	<0.05		
	fT4 (0.6-2.3 ng/dL)		>6.60			2.97	1.38		
	T3 (60-90 ng/dL)					86			
	TRAb (<15%)				85.92				

## Discussion

TPP is a rare complication of hyperthyroidism and thyrotoxicosis, which is common in 20- to 40-year-old Asian males. Hot weather, increased exercise, and consumption of large numbers of high-carbohydrate meals and alcoholic drinks are typical causes [8,9]. TPP most commonly presents as rapid-onset, transient, symmetrical muscle weakness and fatigue that are associated with hypokalemia [10]. Excessive thyroid hormones can directly alter the cell membrane permeability to potassium by increasing Na+/K+ ATPase activity that induces massive abnormal potassium influx [11]. Hyperthyroidism has also been reported to produce an increased sensitivity to beta-adrenergic stimulation of the gland and an increase in the number of beta-adrenoceptors [12]. This hyperadrenergic state may lead to the activation of the Na+/K+ ATPase, resulting in an increased cellular influx of potassium [13]. Therefore, almost all primary symptoms are related to the change in the potassium level.

Here, we report the presence of rare profound hypokalemia in a patient who was diagnosed with first-time onset of TPP. To investigate how the patient tolerated the severe hypokalemia without experiencing critical symptoms (such as chest pain or altered consciousness), we exhaustively evaluated the patient’s medical history and lifestyle habits. Finally, we found that he had a history of chronic alcohol abuse (an 1-L bottle 45%-48% of liquor per week) for more than 10 years. Although in the previous studies of TPP some patients were reported to have a history of alcohol consumption [14-16], but no patients with asymptomatic profound hypokalemia similar to our case were identified. Symptoms of the patient in the previous studies even included chest pain [17]. Thus, we suggest that chronic alcohol abuse may be an important factor that induced chronic hypokalemia.

Hyperthyroidism can induce hyperadrenergic conditions by a chemical structure similar to that of catecholamines, activation of Na+/K+ ATPase, and increase of the sensitivity of ꞵ-receptors [18,19]. Moreover, chronic alcoholism is usually associated with electrolyte abnormalities. The causes of hypokalemia in chronic alcoholism include gastrointestinal loss (diarrhea or vomiting), hypomagnesemia, and renal tubular dysfunction [3]. There are few studies of the association between alcohol consumption and thyroid function in individuals who currently actively consume alcohol, because many studies are conducted in detoxification programs. However, the effect of chronic alcohol consumption on hyperthyroidism may be considerable [20]. In this case, chronic alcohol abuse may have increased the patient’s tolerance to the profound hypokalemia such that it did not immediately show critical symptoms such as GI symptoms. This might be dangerous for patients because it may delay the diagnosis and crucial medical treatment.

Therefore, according to this case report, we suggest that chronic alcohol consumption or abuse may lead patients, especially those with hyperthyroidism, to ignore or delay treatment.

## Conclusions

Early diagnosis of TPP is important in the case of a patient presenting with hypokalemia and hyperthyroidism. To avoid complications and mortality of TPP in patients with hyperthyroidism, lifestyle changes such as ceasing alcohol consumption should be recommended.
